# ‘It’s Already Hard and It’s Nearing Impossible’: A Thematic Analysis of Submissions by Rural Veterinarians to the NSW Parliamentary Inquiry into the Veterinary Workforce Shortage

**DOI:** 10.3390/vetsci12010069

**Published:** 2025-01-17

**Authors:** Sharon Mary Pepita Thio, Anne Quain

**Affiliations:** Sydney School of Veterinary Science, University of Sydney, Camperdown, NSW 2006, Australia

**Keywords:** veterinary workforce, parliamentary inquiry, rural practice, rural veterinarians, workforce feminisation, reflexive thematic analysis

## Abstract

There is a global veterinary workforce shortage, particularly in rural and regional areas. In response to this shortage in New South Wales (NSW), the State Parliament launched a Parliamentary Inquiry in 2023. The Inquiry received 205 publicly available written submissions, providing insights into the perceived challenges and barriers for veterinarians working in rural practice, and how the perceived impact of the feminisation of the workforce interacts with this. By analysing a subset of submissions written by veterinarians reporting lived experiences in rural practice (*n* = 63), we identified eight major themes. These were as follows: rural practices are not financially sustainable; rural veterinarians often have a more challenging and higher workload than their urban counterparts; working in rural practice increases challenges to health and wellbeing; it is difficult to recruit and retain people in rural practice; veterinary students are poorly selected and not well prepared for rural practice; clients have unrealistic expectations of rural veterinarians; rural practice is not compatible with family life; and veterinarians have mixed opinions regarding whether an increase in the proportion of female veterinarians is a key contributing factor in the shortage of veterinarians. These findings may assist in the development of recruitment and retention strategies for rural veterinary practice.

## Simple Summary

There has been increased attention on the global shortage of veterinarians, which can especially impact rural and regional areas. In response to the shortage in Australia, the New South Wales (NSW) State Parliament launched a Parliamentary Inquiry in 2023, which invited submissions from various stakeholders. Our aim was to explore the challenges and barriers reported by veterinarians in rural practices. We also sought to understand how the increased proportion of females in the veterinary workforce was perceived to affect the shortage in rural areas. Eight major themes were identified in our analysis: rural practices are not financially sustainable; rural veterinarians often have a more challenging and higher workload than their urban counterparts; working in rural practice increases challenges to health and wellbeing; it is difficult to recruit and retain people in rural practice; veterinary students are poorly selected and not well prepared for rural practice; clients have unrealistic expectations of rural veterinarians; rural practice is not compatible with family life; and veterinarians have mixed opinions regarding whether an increase in the proportion of female veterinarians is a key contributing factor in the shortage of veterinarians. These findings may assist in the development of recruitment and retention strategies for rural veterinary practice.

## 1. Introduction

There has been growing concern regarding a global veterinarian workforce shortage, particularly in the last five years [1,2,3,4,5,6]. This represents an alarming post-pandemic trend around the world, where an increase in demand for veterinary services associated with increased companion animal ownership has worsened staff shortages [7]. The problem is thought to be exacerbated in Australia due to both its significant animal agriculture sector and high rates of companion animal ownership [8,9]. It was reported that in 2023/2024, 37% of veterinary job vacancies in Australia took more than 12 months to fill or remained unfilled [10]. The shortage is widely felt across different sectors, including clinical practice, research, academia, industry, and government.

Veterinarians are not just clinicians, but also play critical roles in maintaining animal welfare and public health, undertaking both opportunistic and targeted disease surveillance [11,12]. As a result, a workforce shortage can have severe negative impacts on animal health and welfare, as well as negatively impacting the health and wellbeing of humans who depend on them, and the income of animal-based industries.

Compared with urban populations, rural, regional, and remote areas appear to be disproportionately impacted by the veterinary shortage, partly due to the greater spatial distribution of veterinary services and increased economic reliance on large animal production in those regions [2,13]. While the supply of veterinarians has increased in an almost linear fashion in the Anglosphere due to the opening of more veterinary schools, local shortages in rural areas have not necessarily improved [14]. Although the 2003 Frawley Report into rural veterinary services found there was no overall shortage of veterinarians in Australia at that time, it acknowledged that rural veterinarians were vulnerable to local staff shortfalls that could potentially extend into chronic shortages, as the availability of veterinarians was dependent on their willingness to live in those areas [15]. More than 20 years later, supply issues of veterinarians in rural areas have only worsened.

Despite the obvious need for veterinary services in these regions, rural veterinary service providers still struggle with the recruitment and retention of staff [16]. In a White Paper on the Veterinary Workforce, former Australian Veterinary Association (AVA) President Dr Bronwyn Orr wrote that the “shortage in most countries is likely a distribution problem…rather than a true shortage…”, where “certain segments of the industry cannot attract or retain veterinarians” [17]. In 2023, regional areas were more affected by protracted job vacancy times than metropolitan areas, with 44% of veterinary job vacancies in regional areas taking more than 12 months to fill or remaining unfilled compared to 28% in metropolitan areas [10]. A previous Australian study found that risk factors for leaving rural practice included on-call and after-hour demands, a low rate of return for hours worked, pursuit of personal career opportunities, lack of employment opportunities for family members, and lack of proximity to friends and family [18]. These concerns are consistent with reports from other countries that face rural shortages. Veterinarians in the United States of America (U.S.) reported similar reasons for leaving rural practice, including emergency duties, lack of time off, low salary, a poor practice atmosphere or culture, and family concerns [19]. The Federation of Veterinarians of Europe attributed the shortage in rural areas to harsh working and living conditions, as well as difficulty in maintaining profitable practices [2].

It has been argued that one factor reported to be associated with challenges in recruitment and retention is the increased proportion of female veterinarians in the profession [10,20,21]. Workforce surveys have shown that female veterinarians are less likely to report full-time employment than male veterinarians, which may contribute to a relative workforce shortage [22]. One explanation is that female veterinarians may have career gaps or switch to part-time employment to have children and take care of them [23,24], though this does not apply to all women. However, there is currently a lack of research into how significantly the feminisation of the workforce contributes to the rural veterinary shortage.

One study attempted to identify structural barriers to rural veterinary practice that could impact female veterinarians by interviewing seven female veterinarians working in rural areas in the U.S. [20]. Female veterinarians reported the historical structuring of rural veterinary practice, establishing a rural client base, and occupational risk factors as challenges that prompted consideration of leaving rural veterinary practice. A previous Australian study using thematic analysis revealed that veterinarians left clinical practice due to a complex combination of personal and work-related factors [25]. There is little research exploring the challenges and barriers to rural practice from the perspective of veterinarians working in these areas. Understanding the challenges and barriers to recruitment and retention of rural veterinarians is the first step in addressing them. Additional studies of the experiences of rural veterinarians are needed to not only interrogate these factors further, but to better understand the nuances of these challenges and barriers within the context of rural veterinary practices.

In June 2023, the Australian State Parliament of New South Wales (NSW) launched a Parliamentary Inquiry into the veterinary workforce shortage [26]. The Inquiry invited written and spoken submissions from a wide range of stakeholders, including veterinarians working in rural and regional settings.

Our aim was to examine the public submissions to the NSW Parliamentary Inquiry and identify perceived challenges and barriers for veterinarians working in rural practice in NSW, and perceived barriers to recruitment of additional veterinarians. In addition, we sought to understand how respondents believed that gender impacted the experience of working as a veterinarian in rural areas.

## 2. Materials and Methods

The NSW Parliament established an inquiry to “inquire into and report on the veterinary workforce shortage in New South Wales” on 9 June 2023 [27]. As part of this inquiry, members of the public, including veterinarians, were invited to make written submissions if they wished to do so, expressing their views to inform the Committee. Parliamentary guidance regarding submissions states that a submission “…may contain facts, opinions, arguments, suggested solutions or tell of your personal experience” [28].

Written submissions were made to the NSW Parliamentary Inquiry between 9 June and 21 July 2023. Unless a respondent requested that the Committee treat a submission as confidential, or the submission was irrelevant to the Inquiry, contained offensive remarks, or was otherwise inappropriate [28], each submission was published online by the NSW Parliament [26,29]. Although these submissions were publicly available, permission was sought and granted by the Chair of the Committee to use the retrospective dataset for this study (M. Banasiak, pers. comm., 24 August 2023). Due to the volume of submissions, other materials available on the website, such as the hearing transcripts and answers to Parliamentary questions on notice, were excluded from this study.

All publicly available, written submissions were downloaded and scanned individually by the first author (S.T.) before a brief quantitative summary was conducted (Table 1). The submissions were first arranged into broad categories of stakeholders, including ‘veterinarians’, ‘others in the agriculture/veterinary sector’, ‘universities’, ‘members of the public’, ‘organisations’, and ‘government’.

**Figure 1:**
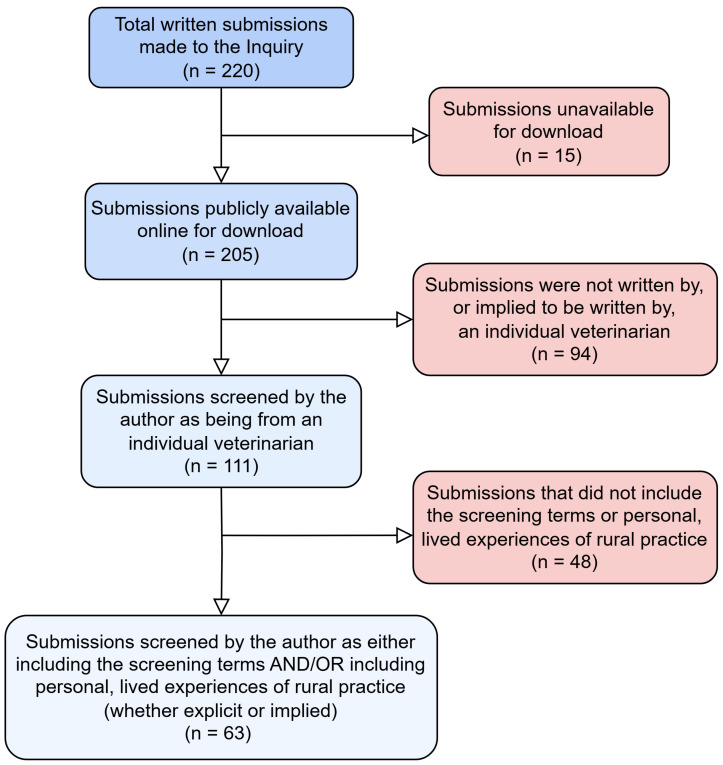
A flow chart of the exclusion and inclusion criteria that were used to screen submissions from the NSW Parliamentary Inquiry into the veterinary workforce shortage.

Selection criteria were then applied to the dataset, which aimed to only include submissions that appeared to be written by individual veterinarians describing experiences in rural practice, as these were more likely to provide insight into lived experiences (Figure 1). For the purposes of this study, the terms ‘rural’ and ‘regional’ are used to describe areas outside of major metropolitan areas in NSW, Australia. This is in line with the government’s usage of the terms in the Inquiry [29].

Submissions made by individual veterinarians on behalf of a larger company or organisation were excluded because they were not likely to describe personal, lived experiences.

Out of the submissions identified to be written by individual veterinarians, the data were screened using the following screening terms: ‘rural’, ‘regional’, ‘remote’, ‘country’, ‘farm’, ‘mixed’, ‘large’, ‘production animal’, ‘livestock’, ‘outback’, ‘bush’, and ‘small town’. The first author also read through the entire dataset to understand the general context and sentiment of the writer of each submission, looking specifically for submissions that included first-hand or personally lived experiences of rural and regional practice. The final subset of submissions was statistically summarised and uploaded onto NVivo^®^ (Release 14.23.3 (61), Lumivero, Denver, CO, USA) software to facilitate reflexive thematic analysis.

Qualitative research is based upon an interpretative analysis of data, which can be influenced by researcher subjectivity. Thematic analysis is an established method used to develop, analyse, and interpret patterns across often large datasets, involving data coding to develop themes [30]. We used reflexive thematic analysis as it recognises and values researcher subjectivity. In reflexive thematic analysis, it is acknowledged that the researcher takes on an active role in constructing meaning from data, which is in turn influenced by how the researcher is situated in the world through their social, cultural, political, and ideological positionings [31]. Therefore, it is necessary for researchers engaging with reflexive thematic analysis to articulate their background and position as part of their methods. To this end, the author performing reflexive thematic analysis (S.T.) is a cisgender female, third-year domestic student of Asian background who is completing a Doctor of Veterinary Medicine degree at the University of Sydney. She has a passion for improving the outcomes of both animal patients and the people who take care of them. Although she was brought up in suburban Western Sydney, the author studied agriculture in secondary education, and as a result, has an interest in working in rural and regional areas. In particular, she is interested in tackling gaps and problems in the veterinary profession, especially mental health issues and the current veterinary workforce shortage. The author collaborating on this analysis (A.Q.) is a cisgender female veterinarian working as both a private practitioner in NSW and a senior lecturer in the Sydney School of Veterinary Science. She has twenty years experience working in veterinary clinical practice in urban and regional areas in NSW, Australia.

Submissions were coded inductively for semantic themes using a realist approach rather than using a pre-existing theoretical framework. Following Braun and Clarke’s method of reflexive thematic analysis, familiarisation with the data took place by reading and re-reading the data [31,32]. Initial codes were identified across the entire dataset before being collated into initial themes. The themes were then developed and reviewed in an iterative process by the first author (S.T.) in collaboration with the second author (A.Q.). The first author kept a detailed reflexive journal to reflect on their assumptions [33]. To show an overview of the dataset and the interrelationships between themes, a thematic map was drawn by S.T.

It is important to note that due to the consideration of parliamentary privilege not applying to any publication outside of the Inquiry, as mentioned by the Chair of the House Committee, the final stage of writing up the report using extracts was altered. To avoid citing submissions directly, any excerpts taken from the submissions illustrating constructed themes were paraphrased. Paraphrased excerpts were cross-checked with the second author (A.Q.). When paraphrasing excerpts referring to suicide, we used language recommended by Mindframe [34]. Identifying information, including names of individuals, veterinary practices, organisations, and geographic locations, was removed.

## 3. Results

In total, there were 220 submissions made to the Inquiry, of which 205 were published on the website [26] and available for download (see Table 1). The word count of the submissions ranged from 18 to 31,271. Selection criteria were applied to obtain the final subset of submissions (see Figure 1).

**Figure 2:**
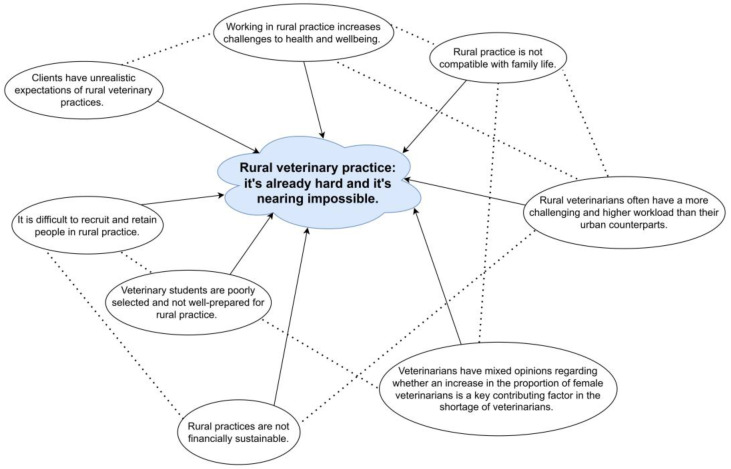
A thematic map depicting themes constructed through reflexive thematic analysis on the challenges and barriers to rural veterinary practice based on submissions to the NSW Parliamentary Inquiry into the Veterinary Workforce Shortage by veterinarians with lived experience of rural practice (*n* = 63). The themes are organised around a central concept. Solid lines depict relationships between themes and a central concept, while dotted lines depict relationships between themes.

**Figure 3:**
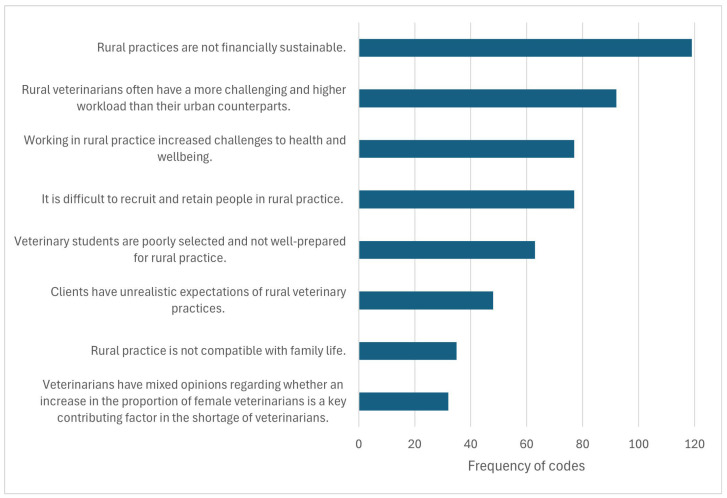
A bar graph depicting the frequency of codes attributed to each theme in a subset of submissions made to the New South Wales Parliamentary Inquiry into the veterinary workforce shortage.

A total of 63 written submissions met the inclusion criteria. The submissions ranged from 70 to 10,094 words in length, with a total of 102,264 words. We described eight major themes, which all related to a central theme of ‘Rural veterinary practice: it’s already hard and it’s nearing impossible’ (see Figure 2). The frequency of codes attributed to each of the themes was also recorded (see Figure 3).

### 3.1. Rural Practices Are Not Financially Sustainable

Numerous respondents reported that it was not financially sustainable to run a veterinary practice in rural areas due to high overheads and low profit margins. They described poor remuneration relative to the complex and challenging nature of work required, long working hours, and after-hours. This was viewed more negatively in light of the large amount of debt veterinary students increasingly accrue. Several respondents reported that their poor remuneration would not allow them to repay this debt quickly, reducing their ability to save and delaying their purchase of a house.

Teaching, mentoring, and training veterinary students and new graduates are often seen as core responsibilities of veterinarians. Some respondents reported that these responsibilities were associated with opportunity if not financial costs for practice owners, for which they received no compensation or support, adding to rural economic pressures. In turn, poor support for new graduates can also contribute to attrition of veterinarians. One respondent described their new graduate experience with a mentor who was unwilling to offer support, to the point where at least five new graduates who had started working at the same practice no longer practised veterinary medicine.

Respondents reported facing multiple threats and barriers to practice profitability and income-earning potential. For example, the rescheduling of previously prescription-only medications increased competition with non-veterinarians for income. Numerous respondents noted growing competition for veterinary-related work between rural veterinarians and lay people (for example, the provision of equine dentistry services by lay people).

There were differing opinions regarding the impact of corporatisation on the financial status of individuals and practice profitability. For some, a corporate buyout provided financial security, but several respondents felt that this ‘exit strategy’ was not available to them due to a perception that corporate companies were less likely to invest in rural practices. Others reported that where corporate companies had bought rural practices in a particular area, practice profitability suffered. One veterinarian explained how a nearby corporate-modelled clinic halted the provision of large animal services, which in turn increased the relatively less profitable, large animal workload of other veterinary practices in the area.

### 3.2. Rural Veterinarians Often Have a More Challenging and Higher Workload than Their Urban Counterparts

Respondents from rural and regional areas overwhelmingly described a more challenging and/or higher workload when compared with practitioners in urban settings. They reported undertaking extensive after-hours and emergency work, with fewer options for relief due to challenges in attracting additional staff or locum coverage. Geographic isolation led to rural veterinarians facing long or ‘unreasonable’ travel distances to their clients, adding to already long working hours and generating logistic problems. Numerous submissions described that providing an after-hours service was an additional burden on top of normal working hours and a feature of the job that they found the least enjoyable. For example, one respondent talked about their experience of working in the clinic from 8 am until 6 pm, being the on-call veterinarian that night from 6 pm to 8 am, and being rostered to work again from 8 am until 6 pm the next day.

Given the reported higher workloads, longer hours, and challenging cases with a relative lack of staff and resources, a number of respondents felt that it was unfair that rural veterinarians were held to the same regulatory standards as their better-supported counterparts. For example, some veterinarians felt that the complaint process to the Veterinary Practitioner’s Board (the independent governing body of registered veterinarians in NSW) favoured the complainant in allowing what they perceived to be petty or vexatious complaints to be investigated, rather than being dismissed. In an effort to avoid facing litigation, many veterinarians felt that they had to spend more time on excessively detailed records, which only contributed further to their workload and stress. One respondent reported that a friend took their own life when informed of a complaint made against them to the Veterinary Practitioner’s Board.

Respondents also described challenges they felt were specific to rural practice. Commonly reported was the obligation to respond to Emergency Animal Disease (EAD) outbreaks despite the fact that many rural veterinarians felt they were not adequately remunerated or resourced to provide this ‘public good’ service. Participation in EAD response was often not required of their urban counterparts due to their distance from disease outbreaks and relative lack of expertise with the species involved.

Another unique challenge was providing back-up veterinary support in the event of complications caused by others. Such complications could arise due to work performed by lay people with inadequate training and skill, for example, those providing equine dentistry services. Respondents also described being ‘expected to’ provide support for veterinary colleagues, such as providing after-hours services for clients of mobile veterinary services that could not provide the full offerings of a ‘brick-and-mortar’ veterinary clinic.

Due to long distances, client cost constraints, and logistic challenges, referrals for intensive or specialist care were not always options. Some respondents felt that this placed additional pressure on rural veterinary practices to manage more complex or challenging cases.

### 3.3. Working in Rural Practice Increases Challenges to Health and Wellbeing

Respondents detailed largely negative impacts of rural practice on both their mental and physical health and wellbeing. They described how the combination of many factors, such as high workloads and client expectations, led to mental fatigue, burnout, mental health issues like anxiety, and suicide. Suicide was a major concern among those who talked about mental health, with several submissions stating that they had personally known people in the profession who had died by suicide. One veterinarian who worked in a regional practice revealed in her submission that her husband, who also was a veterinarian, had taken his own life.

Numerous respondents stated that they faced verbal abuse or pressure from clients which negatively impacted their mental health. Because of their identifiable role in the local community, some respondents reported that interactions with clientele outside work blurred the boundaries between work and personal life, making it hard to ‘escape’ from work. Numerous respondents described feeling an obligation or external pressure to sacrifice their wellbeing for others, whether that be clients or the animals they were treating. Several respondents recounted being interrupted or forced to work during holidays such as Christmas and important celebrations of loved ones like birthdays. One respondent detailed the loss of a personal relationship because of their inability to separate their personal life from work as a rural veterinarian, which led to the veterinarian feeling burnt-out.

Some respondents felt that they were not well supported in their veterinary work, with particular emphasis on a lack of support for new graduates. In one veterinarian’s account of their experience as a new graduate, they described how—despite promises of support from their employer of being supported in a rural mixed practice clinic—they were left alone due to staffing shortages, which exposed them to abuse from clients and exhaustion from overworking due to after-hours. This sentiment was echoed by other veterinarians who commenced their careers in rural clinics, with one stating that they felt they were deceived by the advertising of clinics with ‘new graduate friendly’ labelling.

Two respondents reported encountering racial discrimination while working in regional or rural practices. One respondent who self-identified as an Asian veterinarian working exclusively in large animal services reported experiencing ‘casual racism’ from clients. Another respondent reported overhearing ‘racist’ comments made by a regional practice owner during a job interview, which deterred them from taking up the role. Both respondents believed that racism would turn graduates from diverse backgrounds away from large animal practice, especially in regional and rural areas.

Numerous respondents described being physically injured or threatened with physical violence in their role as veterinarians working at a rural or regional practice. Examples included being threatened by landholders and farmers at gunpoint, being physically assaulted after diagnosing a disease that required property to quarantine, and having a car accident while driving a short distance home after a busy weekend on-call with few hours of uninterrupted sleep. Other physical risks reported included exposure to zoonotic diseases. A veterinarian detailed their frustration with clients who refused to vaccinate their horses against Hendra virus, posing a significant zoonotic health risk to the attending veterinarian. Multiple respondents reported fears regarding personal security, especially when working after-hours alone, with particular concern raised regarding the safety of young female veterinarians.

### 3.4. It Is Difficult to Recruit and Retain People in Rural Practice

Veterinary practices in rural and regional areas found it difficult to recruit and retain staff. Numerous respondents felt that the poor or absent development of infrastructure and services served as a disincentive to move to or stay in rural and regional areas. One respondent described being on a waiting list for childcare for twelve months as a barrier to returning to work following maternity leave. Other respondents mentioned that the housing shortage in rural regions prevented veterinarians from relocating to areas where they were needed, with one respondent stating they had to turn down two regional jobs for this reason. Not only was attracting veterinarians to the regional and rural roles difficult but retaining them was also challenging. One respondent described how many veterinarians were being lost from rural areas due to relocation to major cities or leaving the profession altogether.

Due to the difficulty in attracting new veterinarians to rural practices, retiring rural veterinarians were often not replaced. Several respondents expressed their desire to exit full-time work as a veterinarian but felt unable to do so due to the absence of someone to provide continuity of veterinary care. One respondent, who identified himself as a 1977 graduate, reported that he was ready to retire but unable to fill his position despite advertising the role for over twelve months.

Difficulties in recruiting veterinarians from within Australia led some respondents to conclude that one of their only remaining options was to recruit veterinarians from overseas. However, numerous submissions detailed that the red tape surrounding immigration and registration requirements made it very difficult, if not impossible to secure these prospective employees. Additionally, overseas veterinarians were also considered by some respondents to be ill equipped or not well trained for Australian veterinary practices or standards of animal care.

### 3.5. Veterinary Students Are Poorly Selected and Not Well Prepared for Rural Practice

According to several respondents, a key factor contributing to the workforce shortage was what they perceived to be poor selection and inadequate training of veterinary students in Australia, making them ill suited to rural practice. Numerous respondents blamed universities and their selection criteria, stating that universities do not select the ‘right students’ for entry into the veterinary degree. Some criticised the selection criteria as placing too much emphasis on high Australian Tertiary Admission Rank (ATAR) scores, rewarding academically gifted students at the cost of those with greater emotional intelligence and resilience. Others stated that the university practice of recruiting a greater proportion of international students in veterinary medicine led to a loss of potential graduate veterinarians who would participate and remain in the domestic workforce.

Numerous respondents observed how a perceived emphasis and reliance on diagnostic testing in veterinary curricula was associated with a decline in graduate competencies. Respondents also reported that universities prioritised teaching of ‘gold-standard care’, often inaccessible or unaffordable to owners, as opposed to a ‘spectrum of care’ approach, which may be more accessible and possibly more appropriate for those living and working in rural areas. Universities were also blamed for not adequately training students in emotional intelligence, resilience, and general confidence, with one respondent believing that many students were not being empowered enough to become competent and confident clinicians, instead being led to second-guess themselves in tasks that were realistically achievable.

Some respondents expressed concern that veterinary students had unrealistic expectations of rural and regional practice. One veterinarian described how new graduates expected to be walked through problems and scenarios before they faced them themselves. Others commented how ‘work–life balance’ and lifestyle seemed to hold greater value to veterinary students and graduates, who were perceived to be disinterested in owning their own practice, working full-time, or performing after-hours duties.

### 3.6. Clients Have Unrealistic Expectations of Rural Veterinarians

Numerous respondents reported what they perceived to be unrealistic expectations that clients and the public have of veterinarians, particularly those in rural and regional practices. Not only were veterinary clients and community members generally unaware of the variety and extent of work that veterinarians undertake, but there were also misconceptions regarding the amount of pay, the after-hours burden, and the costs of providing veterinary services. One respondent claimed that television shows such as *Bondi Vet* showcased the positive side of veterinary medicine without depicting the financial costs of veterinary care as well as the skill and work required to manage those cases. They reported that this inevitably painted an unrealistic picture of the veterinary profession. Other respondents attributed unrealistic client expectations regarding the costs of veterinary care to the general public’s ignorance of the true costs of human healthcare, which is heavily subsidised by the Australian Government through Medicare.

### 3.7. Rural Practice Is Not Compatible with Family Life

Multiple respondents stated that rural veterinary practice was not compatible with having and raising a family, as veterinarians were unable to enjoy time with their partners, children, or extended family due to work. For example, one respondent reported missing the first night of a weekend away with her husband and children because her evening shift extended well beyond her scheduled finish time. Another respondent reported how he had planned to leave for a holiday with his wife and children but was interrupted by a call-out to a cow suffering from dystocia, delaying their departure. After spending hours attending to this animal, the veterinarian returned home to find his veterinarian wife performing emergency surgery on a dog, further delaying their departure and reducing their holiday time. Similar stories appeared in other responses, with one respondent expressing his regret of missing out on his children growing up, unable to go away for weekends or attend important family events such as birthday celebrations and school functions.

On top of the previously mentioned challenges of securing childcare services in rural areas, some respondents also reported difficulties in negotiating maternity leave with their workplace. One respondent stated that many veterinarians who returned from maternity leave were given the choice of either going back to full-time work or leaving the practice, which often resulted in pushing veterinarians out of clinical work entirely and into other jobs with more flexible hours to facilitate childcare. One respondent, who self-identified as a practice owner, suggested that a key factor behind employees returning only on a part-time basis following maternity leave was difficulties experienced in finding childcare places, especially for those who lack family support for dropping-off and picking-up children to and from childcare in rural and regional areas. Several respondents who remained in clinical practice while trying to raise a family reported burnout and poor work–life balance, unable to juggle work demands and family priorities, without any time to recuperate or practice self-care. Rural veterinary practice was hence summarised by one respondent as ‘not very family-friendly’.

### 3.8. Veterinarians Have Mixed Opinions Regarding Whether an Increase in the Proportion of Female Veterinarians Is a Key Contributing Factor in the Shortage of Veterinarians

The increased proportion of female veterinarians compared to male veterinarians in the workforce was a common topic of discussion. Of the 32 responses coded to this theme, 18 took a negative view on an increased percentage of women in the profession, 12 were neutral, and 2 were positive.

For the respondents who viewed the feminisation of the profession negatively or perceived it to impact the workforce in a negative way, varying reasons were given. One respondent who identified as male stated that female veterinarians prefer to stay in urban areas and do small animal work as opposed to country work which they considered to be ‘men’s work’. Multiple respondents perceived that the veterinary school selection process was skewed due to the increased proportion of female veterinary students relative to male veterinary students. For example, one veterinarian expressed their view that ‘young ladies’ were more able to achieve higher grades in high school than male students, whether they be from rural or urban backgrounds. The same respondent argued that the ‘biological clocks’ of women would inevitably mean a significant number of females would take time off for maternity leave, leaving the rural veterinary workforce short-staffed. They added that should these women return to work promptly, work duties would then limit their availability to cater for family life or motherhood.

Some respondents reasoned that though the gender demographics of the workforce had changed to become more female-dominated, there was neither a largely positive nor negative impact on the workforce. One respondent stated that although their staff was mainly female, the issues they faced in regard to after-hours work taking away time from family was not strictly a feminine issue, but would also apply to any male colleague in the same situation. This echoed the sentiment of other respondents who did not necessarily mention the feminisation of the profession as an issue, but rather how balancing family life with veterinary work in rural areas was extremely difficult.

Two respondents viewed the feminisation of the profession as having a positive impact on the veterinary workforce. One respondent believed that the benefits that females offered the profession were frequently downplayed, citing that a woman’s higher life expectancy could lead to them working for longer. Another submission from a respondent who identified as a male veterinarian reported that his wife was also a veterinarian, stating that he believed female veterinarians matched up physically and intellectually to their male counterparts, and described how he believed there were many things that his wife could do that he could not.

It was troubling to read these accounts detailing the challenges of rural practice as a young female veterinary student (S.T.). Many of the experiences highlighted in the submissions were negative and could discourage people from entering the profession. Some respondents blamed vulnerable individuals such as veterinary students, instead of acknowledging the systemic problems that are clearly plaguing the veterinary profession. Hence, reading these accounts proved to be frustrating and demoralising at times.

## 4. Discussion

To the authors’ knowledge, this is the first paper that uses reflexive thematic analysis to explore the perceived challenges and barriers of recruiting and retaining veterinarians, specifically in rural veterinary practice in Australia. We identified eight key themes that were pertinent to the challenges of rural veterinary work and were perceived by respondents to contribute to the veterinary workforce shortage: rural practices are not financially sustainable; rural veterinarians often have a more challenging and higher workload than their urban counterparts; working in rural practice increases challenges to health and wellbeing; it is difficult to recruit and retain people in rural practice; veterinary students are poorly selected and not well prepared for rural practice; clients have unrealistic expectations of rural veterinarians; rural practice is not compatible with family life; and veterinarians have mixed opinions regarding whether an increase in the proportion of female veterinarians is a key contributing factor in the shortage of veterinarians.

Not unexpectedly, these themes align with the NSW Parliamentary Committee’s finding that veterinarians in rural and regional areas face additional challenges to their veterinary counterparties in metropolitan areas. These were listed as follows: “difficulties in providing after-hours coverage, large distances to be travelled to visit clients on farm, difficulty recruiting and retaining veterinarians, particularly for large animal work, difficulty finding a buyer for their practice, difficulty finding suitable housing, a lack of mentors, particularly for newly graduated vets, a lack of engagement with their services by primary producers” [29]. However, the State Parliament’s report necessarily provides a brief summary of the key issues arising in written and verbal submissions made by a range of stakeholders (including non-veterinarians and corporations) across the entire veterinary sector.

Our analysis provides details regarding the day-to-day lived experiences of those who work or have worked in rural and regional veterinary practices, including the current impacts of these challenges on veterinary team members. This includes impacts on the mental and physical wellbeing and personal lives of these individuals, which in turn can impact career satisfaction, quality of life, and career longevity. Policymakers, including governments and regulators, need to ensure that these particular challenges are addressed in order to take effective action to address the shortage of veterinarians in rural and regional areas.

The themes align with previous studies that identified financial insecurity, longer working hours, heavier after-hours burden, increased physical and mental health risks, limited access to services such as healthcare and childcare, and unrealistic client expectations as either unique or greater challenges for veterinarians working in rural and regional areas [35,36,37,38,39,40]. An Australia-wide survey revealed that rural veterinarians were concerned about the low rate of remuneration relative to hours worked, lack of opportunities for career diversification, lack of social activities, and lack of opportunities for family members, such as employment opportunities for spouses and school opportunities for children [18]. A survey of 28 European countries also found that rural veterinarians grappled with the lack of attractiveness of rural areas, the difficulties in setting up a profitable business, harsh working conditions, and the decline of public procurements [2].

While the Parliamentary Committee report recognises the challenges of working in rural practice, our analysis suggests an overriding concern regarding the sustainability of rural veterinary practice. This cannot simply be addressed by solving deficiencies within the veterinary profession, as it encompasses broader economic concerns, including progressive reduction in government subsidisation of rural economies. The economic disparity between rural and metropolitan areas has been a topic of discussion of policymakers and extends beyond the veterinary profession [41,42]. A ‘dual economy phenomenon’ (the existence of two separate economic sectors within one country) is apparent when comparing the different health outcomes of humans in rural versus urban areas [38,43]. Though less-often discussed in veterinary medicine, the dual economy divide between urban and rural Australia impacts rural practitioners. Clients living in rural areas often do not have the same financial resources as those in metropolitan areas, and this directly impacts practice profitability. As a result of socioeconomic factors beyond the control of individual veterinarians or practices, ‘care deserts’ may exist in rural or remote areas where there are few to no veterinary services. This can affect care provision, with a negative impact on the welfare of animals and the wellbeing of humans who depend on them, creating challenges for veterinarians serving large geographical areas [44].

It is important to note that while rural and regional areas tend to be more reliant on agriculture to support local economies, the term ‘rural practice’ is not synonymous with ‘large animal practice’, though there is a greater likelihood that a rural practice will service large animals. Hence, even rural practices with a higher proportion of small animal patients are vulnerable to the impact of the dual economy phenomenon, though rural clients may expect and demand the same standard of care provided by veterinarians located in major metropolitan centres without understanding the relative costs of delivering that standard of care.

### 4.1. Rural Practices Are Not Financially Sustainable

The finding that rural practices are not financially sustainable has been highlighted in previous studies [41,42,45]. Rural practices operate within rural economies, which, according to the dual economy model, are different and separated from more affluent urban economies. This may be why the effects of increased costs of living, in addition to increased costs of operation and provision of veterinary services, are felt more keenly in rural practices. Without government support of rural economies in general, whether that be financially or through other means, this will not change. In the Veterinary Workforce White Paper, former AVA President Bronwyn Orr found that veterinary workforce challenges in rural and regional areas would worsen without direct government intervention. She acknowledged this was unlikely, writing that “given Australia’s love of deregulation in the agricultural industry, there is almost no appetite for market intervention via direct or even indirect subsidies” [17].

The emergence of neoliberalism as a social movement has meant that relationships between the state and the veterinary workforce have changed. As government bodies around the world have increasingly attempted to redistribute responsibilities and costs of animal health, veterinarians have been left without the financial support that they used to receive [42]. An example of one such program in the past was Australia’s Brucellosis and Tuberculosis Eradication Campaign (BTEC), where veterinarians were previously compensated by the Federal Government for taking part in surveillance and control efforts [46]. When a similar program for brucellosis in the United Kingdom (U.K.) was defunded, many clinics lost a vital source of income [42]. Hence, although the national BTEC program in Australia was successful, rural practices that facilitate food animal production and biosecurity have been economically hamstrung without this funding, which likely significantly subsidised their operational expenses. The loss of this source of income in turn means fewer resources to support animal welfare and biosecurity in rural and remote areas.

The agricultural sector in Australia has faced considerable changes in recent years, including a reduction in the number of agricultural businesses over time as average farm sizes have increased due to consolidation [47]. This is symptomatic of a worldwide shift in agricultural management, as countries such as the U.K. also face a decline in family-run farms, the growth of large corporate operations, and a greater number of hobby-style farm enterprises [45]. Consequently, the demand for some large animal services has declined, leaving veterinary practices that rely on large animal services for revenue in a financially vulnerable position [2,13,35]. There has been a decrease in the proportion of mixed practitioners responding to the AVA’s national Workforce Survey. According to the 2023/2024 Veterinary Workforce Survey Analysis Report, “if this trend continued in future surveys it would support the anecdotal rhetoric that maintaining mixed practice viability with the current veterinary business model is becoming unsustainable” [10].

### 4.2. Rural Veterinarians Often Have a More Challenging and Higher Workload than Their Urban Counterparts

Without large emergency clinics nearby providing 24-h care, veterinarians in regional and rural areas must provide continuous on-call services. Our analysis aligns with previous research that suggests that rural practitioners work longer weeks and are on-call to a significantly greater extent than urban practitioners [18]. A cross-sectional survey of rural and non-rural veterinarians in the U.S. (*n* = 2195) found that rural practitioners frequently reported working over 40 h per week, with a greater amount of time spent on-call [48]. According to the AVA’s most recent Veterinary Workforce Survey, callback hours were highest for equine and mixed practice, with a median of 5 callback hours per week compared to a median of 1 h for “production predominate” practices and nil hours for “small animal predominate” practices. In Australia, mixed practices in particular are more likely to be located in rural areas [10].

On-call duties and after-hours work have been shown to have a detrimental effect on a veterinarian’s job satisfaction, wellbeing, and personal relationships [36]. Negative working conditions, such as long working hours, staffing issues, work–life spillover, and heavy workloads were the most frequently reported stressors in a qualitative Australasian study of veterinary professionals [49]. These were also reported to be common issues by the respondents in submissions to the Inquiry.

The prolonged and unresolvable work stress caused by the mismatch between a consistently high and challenging workload and the resources of a veterinarian can lead to burnout [50]. An Australian study found that longer working hours and having on-call duties significantly increased the likelihood of leaving clinical practice [51]. This effect is likely exacerbated in rural practices with higher on-call demands than those in metropolitan areas.

Veterinarians in rural and regional areas are often responsible for the detection and management of diseases that may pose threats to animal and human health, particularly those that might impact food security and trade. As these concerns primarily involve production animals, veterinarians working in rural and regional areas are expected to be prepared for and engage in response to EADs. Such work is usually performed on top of existing duties and inequitably distributed across the veterinary workforce, with 70% of those in mixed practice and 86% of those in equine practice actively participating in local animal disease management, in comparison to 42% of those in companion animal practice [52].

### 4.3. Working in Rural Practice Increases Challenges to Health and Wellbeing

Our analysis revealed major challenges to the health and wellbeing of veterinarians working and living in regional and rural areas. As previously mentioned, the often more challenging and higher workload that rural veterinarians face in comparison to their urban counterparts, in combination with difficulties in keeping rural practices sustainable, often puts insurmountable pressure on these individuals, as well as strain on personal and family time. This can lead to poorer health and wellbeing outcomes for veterinarians in rural areas.

Veterinarians are frequently exposed to a wide range of hazards that can cause injuries as well as health and safety incidents. Common occupational incidents include sharps-related injuries, animal bites, and zoonotic diseases [53]. In one Indian study, large animal veterinarians faced more physical challenges and were more likely to suffer serious injury than small animal veterinarians, facing over twice the risk of injury compared to their small animal counterparts [37]. Additionally, large animal veterinarians were more likely to sustain a significant injury that results in hospitalisation or multiple days off work, which could add increased stress to the veterinarian, especially if they work in an isolated or rural area [54].

Respondents discussed the links between an increased workload and poorer outcomes for mental and physical health. High workloads and long working hours are known risk factors for suicide, while job-related stress is significantly associated with increased work-related accidents [55,56]. Fatigue in the form of sleep deprivation and insufficient rest is also thought to play a significant role in these incidents, and can lead to deleterious effects on job performance and patient safety due to an increased rate of errors [57,58]. It is also well known that fatigue increases the risk of motor vehicle accidents, which is relevant as a number of respondents reported long-distance car travel as part of their daily work [59].

Some respondents raised concerns regarding discrimination, including racism and sexism. People from typically marginalised groups may be less comfortable working in rural areas: not just people of different cultural backgrounds or race, but also those who identify as Lesbian, Gay, Bisexual, Transgender, Queer, Intersex, Asexual, or other (LGBTQIA+); live with a disability; or are part of other marginalised groups. Those who identify as a member of the LGBTQIA+ community are already at greater risk of negative mental health outcomes, with non-heterosexual, transgender, and non-binary veterinarians having a higher rate of suicidal ideation and attempted suicide compared to veterinarians in general [60]. Hence, these individuals, and others in marginalised groups, may face greater challenges working in rural practices. According to the latest AVA Veterinary Workforce Survey (*n* = 2187), 12.6% of respondents identified as LGBTQIA+ while 4.8% preferred not to say; 37.9% identified as having a disability, chronic condition, or neurodivergence; and 4% did not speak English as their primary language [10]. Thus, experiences of and concerns about discrimination potentially impact a sizeable proportion of the veterinary workforce.

While a tight-knit local community that is commonly found in rural areas can be socially beneficial, veterinarians living in these areas were often unable to escape from clients outside of work. Because of their visibility in the community, rural veterinarians reported feeling under pressure to provide services while ‘off duty’. This could be due to the blurring of the boundary between work and life that being on after-hours duty can cause. Hence, public perception of veterinarians may have a greater impact on the mental health of rural veterinarians when outside of the veterinary clinic than previously recognised.

Our findings conflict with a cross-sectional survey conducted in Germany (*n* = 999) which found that work location did not significantly influence the wellbeing of veterinarians [61]. A cross-sectional survey of rural and non-rural veterinarians in the U.S. (*n* = 2195) reported that rural veterinarians felt more at home, more connected, and more interested in their communities than their non-rural counterparts [48]. Furthermore, rural veterinarians were more likely to report that they liked where they practiced, would be sorry or regretful to leave the community, and felt their community was part of their personal history than non-rural veterinarians.

However, wellbeing can be influenced by a variety of factors, not all of which were accounted for in these studies. For example, in the survey of veterinarians in the U.S., non-rural veterinarians were more likely to report satisfaction with their ability to earn an adequate income, available medical and healthcare services, local shopping, community appearance, recreation facilities and programs, and daycare than their rural counterparts [48]. Additionally, since these surveys were conducted in other countries, it may have been that the results were affected by smaller distances and lesser disparities between rural and urban areas, in comparison to Australia’s large land mass and sparse population distribution.

### 4.4. It Is Difficult to Recruit and Retain People in Rural Practice

Previous studies suggest that rural practices had difficulties in recruiting and retaining staff. A survey of veterinarians in the U.S. found that only 2.7% of respondents who worked as veterinarians in rural practice left due to retirement, while over 50% of respondents left rural practice to pursue careers in urban practice or academia [19].

One factor may be the size of the practice, with those in rural and regional areas likely to employ fewer veterinarians than those in urban settings. This was highlighted in the AVA’s Veterinary Workforce Survey, which found an inverse relationship between the size of the practice and the length of time taken to fill vacancies in regional areas. Thus, 70% of vacancies in practices with one full-time-equivalent (FTE) veterinarian took 12 months or more to fill, compared to 44% in practices with 2–5 FTE veterinarians, and 18% in practices with 6–10 FTE veterinarians [10]. One reason for this, as suggested by the AVA, is that there is less scope to share an after-hours workload with fewer veterinarians. Other possible reasons include less flexibility with rostering (less opportunity to swap or have someone cover shifts) and more limited opportunities for collegial support.

Veterinarians considering working in rural areas may have less choice regarding their working conditions. According to former AVA President Dr Bronwyn Orr, “a veterinary professional in the city has far more choice over where, how and when they work. They can refuse to do on-call and after-hours, work reduced hours, treat only one species or perform limited duties and they can ask for higher compensation due to more clinic competition” [17].

Due to the higher proportion of large animal work and higher rates of serious injuries with large animals, there may be a perception that rural work is a ‘young person’s game’. However, the dual economy phenomenon means that new graduates, who are often young and at the beginning of their professional careers, may not necessarily be attracted to non-metropolitan areas due to reduced infrastructure in rural locations, such as fewer options for childcare, fewer opportunities for partners of veterinarians to find appropriate work, and less growth from rural real estate [40,62]. While a cross-sectional survey of veterinarians in the U.S. reported no difference in intent to stay or leave practice between rural and non-rural practitioners [48], the availability (or not) of local amenities including healthcare, recreation, and childcare may play a role in attracting or deterring veterinarians to work in rural practice.

People living in rural areas have limited access to essential services such as healthcare. It has been well documented that in comparison to their urban counterparts, individuals and families living in rural areas have worse physical and mental health outcomes [38]. This may be a particular consideration for veterinarians who have dependent family members or others to whom they provide care. Improvement of working and living conditions has been suggested as a means of improving recruitment and retention of professionals in rural areas [63].

### 4.5. Veterinary Students Are Poorly Selected and Not Well Prepared for Rural Practice

Many respondents believed that the veterinary workforce shortage was caused in no small part by the poor selection and inadequate training of veterinary students for rural practice, which leads to low recruitment and retention of new and recent graduates. This was attributed to many factors, including not selecting candidates who are more likely to develop a career interest in rural veterinary practice, such as students from rural and farm backgrounds [64]. Having friends and family in rural areas or being familiar with the community may also increase the chance of veterinarians staying there or returning to work.

Numerous submissions criticised veterinary school selection criteria as they perceived that veterinary schools placed too much emphasis on high academic marks, rewarding academically gifted students at the cost of those with greater emotional intelligence and resilience. There was a common perception among some respondents that being academically gifted and having a high capacity for emotional intelligence were mutually exclusive.

Some universities have sought to address the perceived shortage of rural veterinarians through rigorous admission criteria and selection processes that favour the recruitment of students with rural backgrounds. For example, Charles Sturt University in regional NSW is one such university that prides itself on its focus on increasing rural veterinary employment, with most graduates from the first two graduating years of the veterinary program still practising in rural locations 5 years after graduation [65].

Veterinary degrees are expensive to deliver and expensive to undertake in comparison to other degrees. Furthermore, a disparity exists between domestic and international student fees. In 2023, it was estimated that domestic students with Commonwealth funded places graduated with a debt of approximately AUD 70,000 to AUD 80,000, while full fee-paying students (domestic or international) were estimated to accumulate a debt of up to AUD 300,000 by graduation [29]. Student debt was considered for the first time in the 2023/2024 AVA Veterinary Workforce Survey, which found that 62.4% of veterinarians had debt at graduation, with a mean of AUD 56,000 for females and AUD 39,000 for males [10]. Course fees are in addition to other costs that students incur, including travel and accommodation expenses when undertaking mandatory placements. In recognition of the financial hardships that placements impose on higher education students, the Federal Government introduced the Commonwealth Prac Payment Scheme to support students undertaking mandatory placements from July 2025 [66]. However, veterinary students were excluded from the list of eligible professions, exposing them to ‘placement poverty’ [67]. The increased intake of international students in some Australian veterinary schools has been partly attributed to the need to offset the financial pressures of Australian veterinary schools and faculties, as the veterinary course is among the most expensive, if not the most expensive course, to deliver [29]. As a result, increasing the proportion of international veterinary students was viewed by some respondents in their submissions to be unhelpful to the veterinary workforce shortage as there is a perception that many of these students return home overseas, leading to a deficit in graduates in the domestic workforce. The Federal Government has announced caps on international students from 1 January 2025 [68], though it is unclear how these caps will impact veterinary schools and faculties within these universities.

Many respondents provided viewpoints and experiences that could unintentionally deter prospective graduates, even those initially wanting to work in rural practice, as the submissions mostly focused on the negative aspects of the profession. Numerous respondents criticised the selection and training processes of universities, which could be disheartening to veterinary students who work hard and sacrifice a great deal to enter very competitive veterinary degree programs. Additionally, many schools such as the one at Charles Sturt University do tailor their selection criteria in order to preferentially recruit students from a rural background [69].

It should be noted that veterinary students have been shown to have higher psychological distress compared to practising veterinarians, medical students, and the general public [70]. Therefore, care should be taken to ensure that the blame for complex, multifactorial challenges is not framed as a reflection of the incompetence or inadequacy of incoming veterinary students, who are the future of this profession. Rather, the focus should be on the structures that are required to support and upskill them.

### 4.6. Clients Have Unrealistic Expectations of Rural Veterinarians

It has been reported that clients and the general public have increasingly unrealistic expectations of the veterinary profession as a whole, especially as there appears to be a widening gap between veterinarian and client perceptions of their interactions [29,71,72]. Rural areas have a greater reliance on the success of veterinary services due to the dominance of agricultural businesses in those regions, which puts more pressure on rural veterinarians and their work. This is further compounded by the increase in client expectations regarding the standard of care of small animals, leading to the feelings of desperation and fatigue that many rural veterinarians discussed in their submissions.

Rural veterinarians reported that clients expected a high standard of care, continuous after-hours care, and participation in EAD and other emergency responses, without appreciating the financial and personal costs of delivering that care with fewer resources than veterinarians based in urban centres. Pet owners and farmers today are often informed and are able to acquire information on animal health from sources other than veterinarians, which can increase or skew their expectations of the service and advice they receive [73]. Although client relationships can be a source of fulfilment for veterinarians, they can also be a source of stress, for example, when clients are abusive or have financial constraints that may impose limitations on animal treatment [49]. A Canadian study that assessed the relationship between veterinarian mental health and client satisfaction found that relatively higher client satisfaction was unexpectedly associated with poor mental health for veterinarians [74]. Pet owners can have expectations that may be difficult to fulfil, including expecting the veterinarian to ask the right questions, and for information to be explained, presented up front, available in various forms, and then to be presented with a range of options, not all of which may be available in rural practice settings. They also anticipate that veterinarians listen carefully and remain respectful of their decisions [39]. Fulfilling all these expectations in the context of a high workload with significant time and cost constraints is often not possible. Some respondents suggested public awareness campaigns to help manage expectations about veterinary care. Organisations such as the AVA have created educational material regarding the costs of veterinary services; however, the efficacy of these programs and campaigns in changing client expectations remains unknown [75].

### 4.7. Rural Practice Is Not Compatible with Family Life

There was a general sentiment that practising as a rural veterinarian was frequently not compatible with domestic and family life. This theme interacts with several of the aforementioned themes, as many respondents felt that the high workload as well as the unique mental health and wellbeing challenges of rural veterinary practice created demands on personal resources that directly competed with those of family life.

Despite increased feminisation of the workforce, traditional gender stereotypes in the veterinary workplace continue to persist, which could exacerbate tension between working as a rural veterinarian and raising a family [76,77]. There remains a stereotype that fathers usually take on the role of the primary earner of the family compared to mothers, who may often reduce working hours to accommodate childcare responsibilities. This heteronormative view is harmful not only to mothers and fathers who do not necessarily fall into these roles, but also to those who it excludes, such as members of the LGBTQIA+ community.

One study showed that female veterinarians who were mothers received significantly more instrumental support from their co-workers, while male veterinarians who were fathers received significantly less support from their co-workers than did men without children [78]. Workplace reinforcement of traditional gender roles and stereotypes may create expectations that are unrealistic or unreasonable, and place undue pressure on individuals, their partners, and their families.

In a cross-sectional survey of rural and non-rural veterinarians in the U.S. (*n* = 2195), 4.2% of the respondents reported family satisfaction as a reason for staying in practice, while 3.6% reported family concerns as a reason for leaving practice. While rural respondents reported greater satisfaction regarding their community as a place to raise a family, the lowest satisfaction among all respondents was with daycare [48].

A lack of resources and services in rural areas affects not only women but men as well, as family is not a single-gender issue, though in the submissions women were disproportionately blamed for the issue by both men and women. Single-income households are becoming increasingly non-viable in the face of rising costs of living [79]. This could also mean people are economically compelled to stay in unhappy or unsafe relationships, further contributing to possible mental health challenges. It is likely that a change in family roles and dynamics over the last century, such as an increased proportion of families in which both parents work, has put more pressure on each member of the family.

### 4.8. Veterinarians Have Mixed Opinions Regarding Whether an Increase in the Proportion of Female Veterinarians Is a Key Contributing Factor in the Shortage of Veterinarians

Feminisation of the veterinary profession is a relatively recent yet rapid development of the workforce that prompted mixed, often strong opinions from respondents. The strength of the views expressed suggests that these may be a source of disagreement and conflict among veterinarians. For example, where employers or colleagues feel that female veterinarians cannot offer the skills or perform the same amount of work as their male counterparts, the women they work with may feel unsupported and unwelcome in practices and therefore may be more likely to leave.

It has been found that although there are now more female than male veterinary graduates, and a higher proportion of women in the veterinary workforce, it cannot be described as feminised [76]. Studies have previously described how the culture of the veterinary profession still values ‘masculine’ work traits, such as full-time work, linear and continuous careers, freedom from familial responsibilities, and work that demands technical expertise over more ‘feminine’ traits such as compassion and communication [76,78]. As a result, female veterinarians may be more prone to mental health issues and are more likely to rely on negative coping styles [80,81]. This is echoed in other related work fields, as a meta-analysis of human healthcare professionals found that burnout is more prevalent in female than male physicians [82]. Another review exploring the stress among veterinarians ascertained that female veterinarians subjectively perceived their psychological workload to be higher than their male counterparts [83].

Many of the respondents who negatively view the increased proportion of women in the veterinary workforce placed the blame on poor student selection by universities that favoured female candidates from urban areas with highly academic backgrounds. However, one study pointed out that the feminisation in veterinary education was not simply due to an increased intake of female students, but a decline in male graduation and male students’ avoidance of fields dominated by women [84]. Explanations for this decline in male veterinary students have been put forward, including a reluctance of males to enter careers with low or stagnant incomes, loss of autonomy associated with an increase in corporatisation, and a ‘trend effect’ (as more women enter the veterinary workforce, the professional prestige of the traditionally male-dominated occupation decreases) [85]. It has been suggested that a targeted approach may be necessary in order to increase the application rates of male candidates, as gendered perceptions of the veterinary profession can be manipulated through intentional gendered messaging [86].

The feminisation of the workforce is perceived to be a barrier or issue for some respondents, but many failed to grasp how it can also be an opportunity for growth and improvement. A survey conducted by the AVA found that respondents who identified as female made up 69% of the Australian veterinary workforce in 2023/2024 [10]. It is essential to consider how we can utilise the changing demographics of the profession to increase its future viability. Diversity and inclusion in veterinary medicine allow for a variety of perspectives and approaches to work together and solve issues, which could help sustain the profession [87,88].

## 5. Limitations and Future Directions

A key limitation of this study is that the demographic details of the submissions made to the NSW Parliamentary Inquiry were largely unknown. Although some veterinarians identified themselves and the location of their practice, many others chose to remain anonymous. Consistent inclusion of demographic information within the dataset may have aided the analysis of certain themes. For example, identification of respondent gender may have provided additional insights into themes such as feminisation of the workforce and gender roles.

Additionally, the work location of respondents was not consistently identifiable. Hence, it cannot be verified if the submissions were made by individual veterinarians who were indeed within rural or regional areas. While the entries were categorised into stakeholder groups, we cannot be completely certain that all entries were written by individual veterinarians. It is possible that not all respondents included in this study currently work or worked in a rural setting. Nonetheless, most responses were extended and provided rich detail, suggesting at least previous experience in rural practice.

Paraphrased examples do not capture the true essence of the data in the same manner that direct quotations do. Furthermore, paraphrasing may inadvertently introduce bias, reflecting the researcher’s interpretation of the quotes. To minimise potential bias, paraphrasing was cross-checked by both authors.

Submissions to the Inquiry were voluntary, meaning that they do not necessarily represent the views of persons not motivated to submit a response (non-response bias). There is a certain expectation that a Parliamentary Inquiry will generate recommendations that can lead to policy change. Therefore, people who wish for the profession to be reformed would be more likely to make a submission, and those responses would likely be more critical of the existing state of the veterinary workforce. Those with strong and especially negative feelings regarding the subject matter may have been more motivated to make a submission to the Inquiry. Similarly, the number of individual veterinarian responses was small in comparison to the number of registered veterinarians in NSW, which was 4,628 as of June 2023 [89]. Consequently, responses may not be a true representation of the experiences and views of veterinarians working in rural practice in Australia. To minimise this bias, future prospective studies may include direct personal approaches to participants in randomly selected rural and regional areas throughout Australia. Choice in the format of response (long- or short-form written, participation in focus groups, or individual interviews) or offering an incentive could increase overall response rates [90].

Although this study focused on the broad challenges that veterinarians in rural practices face, it is important to note that there is heterogeneity within rural areas and that in reality, each area faces its own unique challenges. Practices in some regional town centres manage workload successfully by coordinating services, such as local veterinary clinics sharing the after-hours workload by alternating shifts. However, in other rural areas, the community may have only one local veterinary clinic to rely on. Future studies could explore contextual factors responsible for differential severity of veterinary shortages between rural areas. This may serve to identify potential protective factors (such as community characteristics, amenities, or infrastructure) or specific risk factors that could be addressed, which may provide local solutions to the themes presented in this study.

Our study focused on perceived feminisation of the profession, but because of the nature of submissions, we could not explore the intersectionality of gender identity outside of the binary gender system. How exactly gender identity plays a role in the rural veterinary workforce could be explored in future studies.

Due to practical constraints, we had to limit our analysis to discussions of problems rather than solutions. Reflexive thematic analysis of potential solutions discussed in the submissions to the Inquiry would provide rich insights into the types of strategies that veterinarians feel are required to ensure a sustainable rural veterinary workforce. Additionally, this qualitative analysis sets the exploratory foundation for further quantitative research into improving rural veterinary practices.

## 6. Conclusions

The identification of eight major themes describes the current issues of rural veterinary practice: rural practices are not financially sustainable; rural veterinarians often have a more challenging and higher workload than their urban counterparts; working in rural practice increases challenges to health and wellbeing; it is difficult to recruit and retain people in rural practice; veterinary students are poorly selected and not well prepared for rural practice; clients have unrealistic expectations of rural veterinarians; rural practice is not compatible with family life; and veterinarians have mixed opinions regarding whether an increase in the proportion of female veterinarians is a key contributing factor in the shortage of veterinarians. This study provides a useful framework to begin to address current challenges and barriers to rural practice and inform recruitment and retention strategies for rural veterinary practice.

## Figures and Tables

**Table 1:** A quantitative summary of the submissions made to the New South Wales Parliamentary Inquiry into the veterinary workforce shortage.

Number of Submissions (*n* = 220)	
Total number of submissions made to the Inquiry (including supplementary submissions) [29]	220
Number of submissions unavailable for download	15
Number of submissions available for download	205
**Word count characteristics of dataset (*n* = 205)**	
Median word count of submissions	853
Average word count of submissions	1839
Smallest word count of a submission	18
Largest word count of a submission	31,271
Total word count of all submissions	376,885

## Data Availability

The data were analysed for this project with the permission of the Chair of the House Committee, the Hon. Mark Banasiak MP. Submissions, hearings and their transcripts, reports, Government responses, and other documents are available at the following link: https://www.parliament.nsw.gov.au/committees/inquiries/Pages/inquiry-details.aspx?pk=2964.
